# Novel Approach for Lifetime-Proportional Luminescence Imaging Using Frame Straddling

**DOI:** 10.1021/acssensors.4c01828

**Published:** 2024-10-14

**Authors:** Soeren Ahmerkamp, Cesar O. Pacherres, Maria Mosshammer, Mathilde Godefroid, Michael Wind-Hansen, Marcel Kuypers, Lars Behrendt, Klaus Koren, Michael Kühl

**Affiliations:** † Max Planck Institute for Marine Microbiology, 28359 Bremen, Germany; ‡ Leibniz Institute for Baltic Sea Research, Rostock 18119, Germany; § Marine Biological Section, Department of Biology, University of Copenhagen, Strandpromenaden 5, 3000 Helsingør, Denmark; ∥ Aarhus University Centre for Water Technology, Department of Biology, Aarhus University, 8000 Aarhus, Denmark; ⊥ Science for Life Laboratory, Department of Organismal Biology, Program of Environmental Toxicology, Uppsala University, 75236 Uppsala, Sweden

**Keywords:** optical sensors, nanoparticle, planar optode, sensPIV, luminescence lifetime

## Abstract

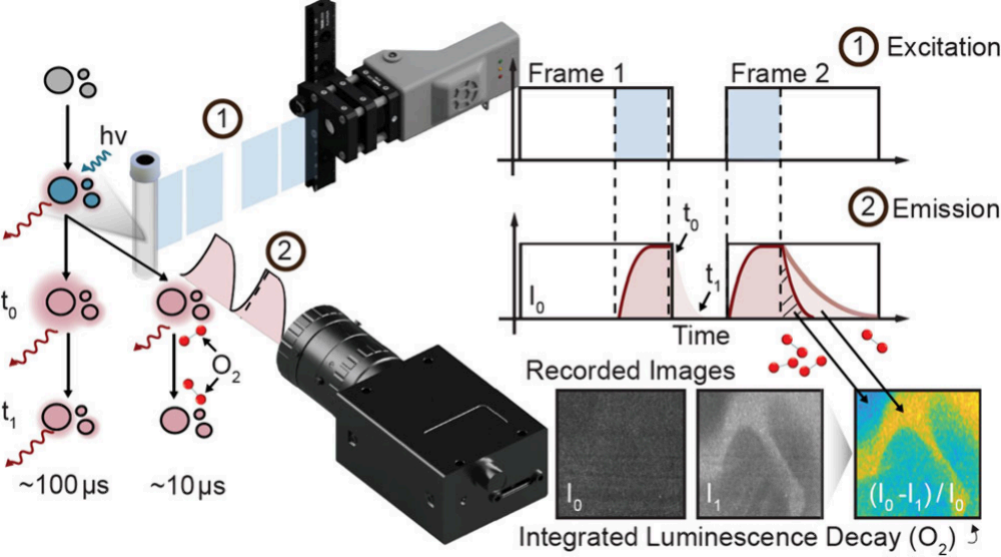

Optode-based chemical imaging is a rapidly evolving field that has substantially enhanced our understanding of the role of microenvironments and chemical gradients in biogeochemistry, microbial ecology, and biomedical sciences. Progress in sensor chemistry has resulted in a broadened spectrum of analytes, alongside enhancements in sensor performance (e.g., sensitivity, brightness, and photostability). However, existing imaging techniques are often costly, challenging to implement, and limited in their recording speed. Here we use the “frame-straddling” technique, originally developed for particle image velocimetry for imaging the O_2_-dependent, integrated luminescence decay of optical O_2_ sensor materials. The method synchronizes short excitation pulses and camera exposures to capture two frames at varying brightness, where the first excitation pulse occurs at the end of the exposure of the first frame and the second excitation pulse at the beginning of the second frame. Here the first frame truncates the luminescence decay, whereas the second frame fully captures it. The difference between the frames quantifies the integral of the luminescence decay curve, which is proportional to the luminescence lifetime, at time scales below one millisecond. Short excitation pulses avoid depopulation of the ground state of luminophores, resulting in a linear Stern–Volmer response with increasing concentrations of the quencher (O_2_), which can be predicted through a simple model. This methodology is compatible with a wide range of camera systems, making it a versatile tool for various optode based chemical imaging applications. We showcase the utility of frame straddling in measuring O_2_ dynamics around algae and by observing O_2_ scavenging sodium dithionite particles sinking through oxygenated water.

The principle of O_2_-dependent luminescence quenching was described >80 years ago,^1^ and optical chemical sensing technologies utilizing this principle have been used for many years.^2−5^ Optical chemical sensing is now used in a wide range of fields, including biogeochemistry, microbiology, biomedicine and biotechnology,^3,6−9^ and is typically based on recording the photoinduced emission of luminescent, analyte-specific sensing dyes that are immobilized in a polymer matrix, as particles or planar foils. For instance, planar optodes enable 2D visualization of concentration gradients of relevant analytes across μm-cm spatial scales, and have revealed intricate microenvironments encountered by microorganisms, plants and animals. Sensor materials for luminescence-based O_2_ sensing have been optimized in recent years resulting in improved reliability, quantum yields and photostability.^10,11^ Further advancements have facilitated the development of sensors capable of detecting a wider range of additional analytes, such as temperature, pH and many others (see (12−14) and references therein).

Upon absorbing photons of specific wavelengths, analyte-specific luminophores experience electron excitation. The energy is quickly dissipated as red-shifted fluorescence or more slowly through intersystem crossing to a long-lived triplet state, resulting in red-shifted emission as phosphorescence (“afterglow”, (1)). The latter is often used for optical O_2_ sensing (e.g., (15−17)), where collisional quenching of the luminophore by O_2_ during the excited state deactivates the triplet state, resulting in reduced emission. This manifests as a decrease in luminescence intensity and decay time with increasing O_2_ concentration, which can be accurately described by Stern–Volmer relations.^18^ Here the luminescence decay or lifetime, i.e., the average duration electrons spend in the triplet state before returning to the ground state, provides a more robust readout for O_2_ concentrations.^19^ The lifetimes of O_2_ sensitive luminophores are typically in the μs-ms range (e.g., (20)).

Chemical imaging with O_2_ optode materials, have camera system-specific challenges. Direct imaging of luminescence intensities are hampered by uneven illumination and excitation, variations in sensor dye distributions, background fluorescence, and effects from photobleaching. Although ratiometric imaging alleviates several of these challenges, luminescence lifetime imaging has emerged as the gold standard for chemical imaging.^21^ However, this technique requires the use of either advanced micro- or macroscopic confocal scanning modules in combination with sensitive, photon counting light detectors^22^ or advanced cameras with fast shutter modulation.^21^ In the latter case, the camera shutter is timed to open during specific time intervals during the phosphorescent decay after an excitation light pulse. From the intensities recorded within different time windows after excitation pulses, the lifetime can then be estimated.^22^ As an alternative to such time-domain lifetime imaging, advanced CMOS cameras also allow for performing similar procedures in the frequency domain.^23,24^ While these imaging modalities offer substantial advantages, they are slow, costly, and complex to implement. Moreover, many of the shutter-modulated CCD camera chips commonly used in these applications are no longer available. There is thus a demand for simpler luminescence lifetime imaging setups to ensure a broader implementation.

In this study, we demonstrate the successful application of a frame straddling technique to record the integral of the luminescence decay curve, which is proportional to the luminescence lifetime of optical sensors. Frame straddling was originally developed for particle image velocimetry (PIV), where it allows for measuring high-speed flows with various inexpensive CCD or CMOS cameras.^25^ In PIV applications, this technique often involves the utilization of double pulse synchronizers with high power lasers or laser diodes, where the pulses are aligned with the camera exposure. Specifically, the initial laser pulse is triggered toward the end of the first image acquisition, while the second laser pulse is triggered at the beginning of the second camera exposure window. In this approach, the effective exposure of the camera is limited to the pulse length and the temporal offset between the pulses determines the maximum flow speeds that can be resolved. Here we demonstrate that this frame straddling approach can be applied to optical O_2_ sensor materials exhibiting concentration-dependent luminescence quenching. The accurate timing of the trigger pulses results in a dimmer and a brighter image, where the difference reflects the integrated, analyte concentration-dependent luminescence decay.

## Results and Discussion

### Theoretical Considerations

**Figure 1:**
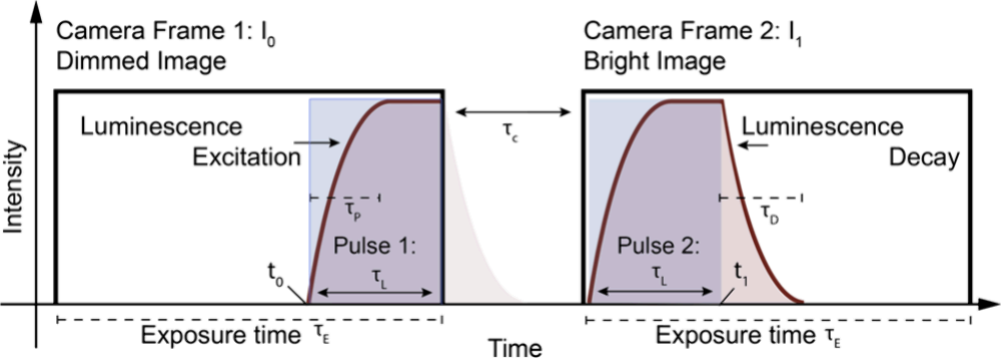
Schematic illustration of the image acquisition timing for quantifying the integrated luminescence decay using the frame straddling technique. The luminescence decay in the first image acquisition is cutoff, while it is fully integrated in the second image acquisition. Blue indicates the emitted laser pulse and red the luminescence emission, while the boxes indicate the exposure window of the camera. *τ_L_*: Trigger length of light pulse, *τ_p_*: Luminescence excitation, *τ_D_*: Luminescence decay, *τ*_E_: Camera exposure, *τ_c_*: Interframe time, *t*_0_: Start of excitation, *t_1_*: Stop of excitation.

During frame straddling two images are recorded: the first image only captures the excitation of the luminescence, whereas the second image captures both the excitation and the subsequent decay of the luminescence. The exposure time of the camera defines the amount of frames-per-second (fps) that can be recorded, while the collected luminescence signal, i.e., the combined sensor luminescence and background fluorescence, is a function of the laser pulse trigger length *τ_L_* (Figure 1).

The luminescence signal change as a function of time, *t*, during the excitation phase, can be expressed via the function:
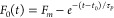
1where *τ*_*P*_ is the characteristic time required for the sensor dye to reach equilibration between excitation and emission within the illuminated field of view, *t*_*0*_ refers to the time the excitation starts. The signal *F_0_* encompasses the emission from all luminophores, i.e., excited electrons may emit photons directly from the singlet state, referred to as fluorescence, or after intersystem crossing to the triplet state, leading to a delayed photon emission, referred to as phosphorescence. As fluorescence is occurring within nanoseconds, it is negligible after the excitation light is switched off, but the average phosphorescence emission occurs on longer time scales and can be expressed as^26^
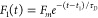
2where *F_m_* is the maximum emission, *t*_*1*_ refers to the time when excitation stops, *τ_D_* is the characteristic luminescence lifetime in the μs-ms range. In case of lifetime-based chemical imaging, *τ_D_* is a function of the analyte concentration. The recorded camera image represents the integrated luminescence signal *F_0_*, where the first frame is
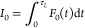
3Here 0 refers to the start of the camera exposure. Effective exposure is only taking place for the duration of the light pulse *τ_L_*. The second frame includes the signal *F_0_* and the additional luminescence emission *F_1_*:
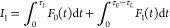
4Importantly, this integration assumes a complete decay of the luminescence signal between the first and second window. When this is not the case, there is an additional contribution from *F_1_* of the first pulse to eq 4, i.e., a carry-over:
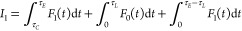
5The interframe time, *τ_C_*, is the camera-specific duration between the capture of two successive frames or images, which depends on the read-out time and clearance of the charges on the camera chip, which is typically 1–10 μs for high-speed cameras and 10 μs to 1 ms for scientific cameras.

Frame-straddling thus records image pairs consisting of a bright image, *I_1_* (eq 5), and a dimmed image, *I_0_* (eq 3). When image *I*_0_ is subtracted from *I_1_*, background fluorescence is effectively eliminated and we obtain the integrated luminescence decay:
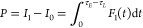
6where *P* is proportional to the luminescence lifetime. If the excitation light field is not homogeneous or the sensor dye concentration is variable, *P* images will not solely reflect the luminescence but also variations in the brightness. In such instances, normalization of the images becomes necessary:
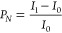
7We note that sensing dyes differ strongly in their luminescence lifetime. In this study, we aimed to resolve the luminescence lifetimes in the range of *τ_D_* = 10–500 μs, which encompasses most optical O_2_ sensing dyes.^12,13^ In this case, the light pulse length should be adjusted to at least *τ_L_* = 50 μs to ensure that (i) the excitation period covers most of the excitation time of the sensing dye, and (ii) enough light is reaching the camera.

We employed eq 4-7 for two main purposes: (i) to estimate theoretical values of the integrated luminescence decay, and (ii) to assess whether the integrated luminescence decay as a function of analyte concentration can be described as a quenching process following the Stern–Volmer relationship. Here we assumed a monoexponential decay with a luminescent lifetime value of *τ_D_* = 62 μs (based on PtTFTPP, see Methods) and an excitation light pulse length of *τ_L_* = 80 μs, which resulted in a calculated integrated luminescence decay of 41%. If the laser pulses are extended to 160 μs, the theoretical value decreases to 20%. These results imply that the brightness of the recorded bright image (*I*_1_) is substantially increased compared to the dimmed image (*I*_0_). To investigate the minimum values of lifetimes that result in a substantial integrated luminescence decay, we gradually decreased *τ_D_* at constant light pulse duration of *τ_L_* = 80 μs. We found that *τ_D_* = 5 μs results in a theoretical luminescence decay of 3%, which we denote as the lower limit of resolvable lifetimes. The luminescence lifetime response to changing quencher concentrations is assumed to follow the Stern–Volmer equation:^27^
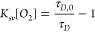
8where K_SV_ is the Stern–Volmer constant, [O_2_] is the quencher concentration and *τ_D,0_* the luminescence lifetime of the sensor dye in absence of the quencher. Using eq 8 in combination with eq 4-6, we found that the integrated luminescence decay generally reflected a lifetime-proportional response to the quencher with Stern–Volmer ratios of P_N,0_/P_N_ ∼ 2–4. However, various effects such as incomplete excitation and carry-over can result in variations of the calibration that depend on the pulse duration and interframe time (Figure S1). These effects are discussed in the following sections.

### Proof of Concept

To test the novel chemical imaging method we used a suspension of styrene maleic anhydride copolymer (PS-MA) sensor particles containing the indicator dye platinum(II) meso-(2,3,4,5,6-pentafluoro)phenyl porphyrin (PtTFPP), which is quenched in the presence of oxygen (O_2_), and the O_2_ insensitive dye Macrolex Yellow (MY) acting as an antenna dye.^28^ The dye mixture in the sensor nanoparticles can be effectively excited around 450 nm. The sensor dye has lifetimes between 22 μs (at air saturation) and 62 μs (at anoxic conditions; see Methods). A 450 nm laser-diode with light-sheet optics (5W; Optolution GmbH) was used for excitation within a pulse duration adjusted to 80 μs, while imaging was conducted with a high-speed camera (Chronos 1.4, Kron Technology), equipped with a 550 nm long-pass filter to only detect the sensor luminescence. Sensor nanoparticles were introduced into two cuvettes; one with air saturated water and one with anoxic water due to addition of the O_2_ scavenger Na_2_SO_3_.

**Figure 2:**
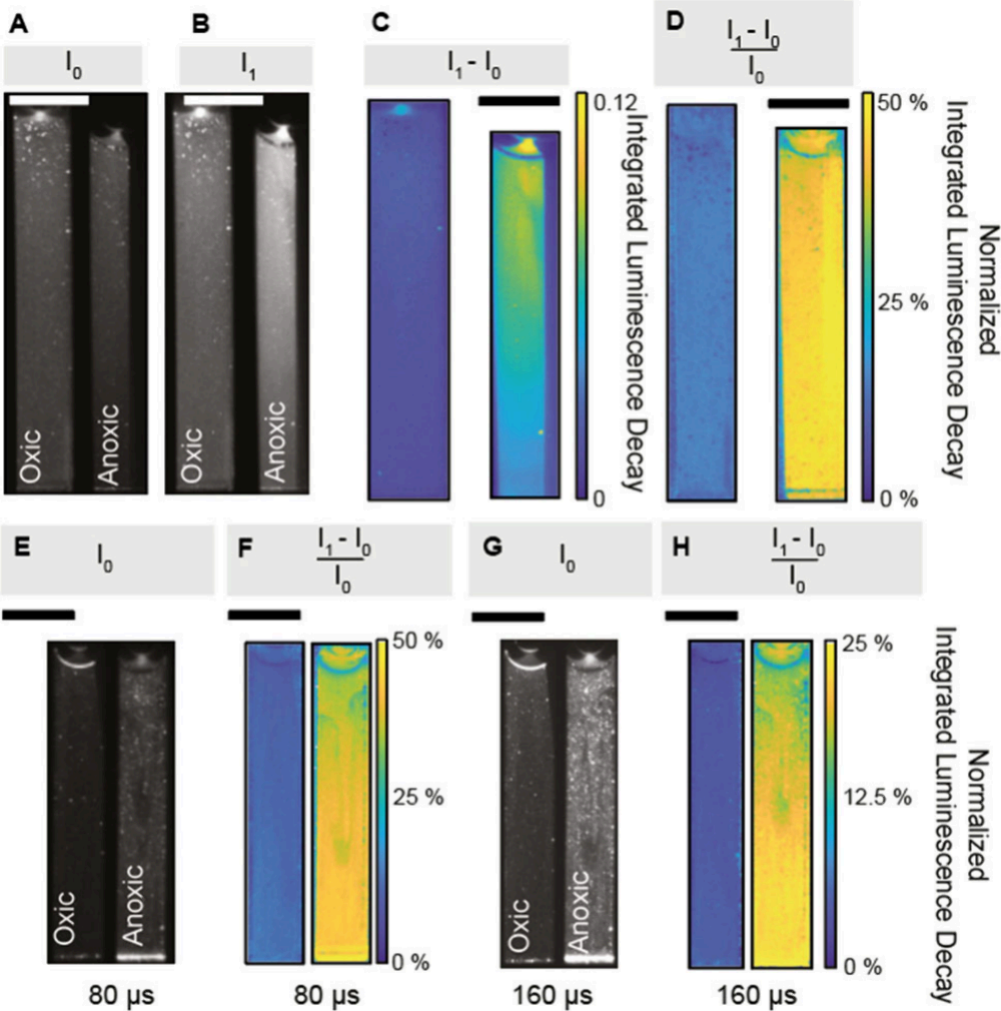
Proof of principle measurements of the integrated luminescence decay using the frame straddling technique for imaging O_2_ in cuvettes filled with a suspension of PSMA sensor nanoparticles containing the O_2_ indicator PtTFTPP and the antenna dye Macrolex Yellow (MY) (ex 450 nm, em >530 nm) in anoxic and aerated water, respectively. The luminescence decay in the first image **A** is cutoff, while it is fully integrated in the second frame **B**. The integrated luminescence decay is directly related to the O_2_ concentration and is calculated from the difference of the two images **C**. To compensate for inhomogeneous illumination and the absorbance of the excitation light, the normalized difference is calculated **D**. In **E-H** different laser pulse lengths were tested with short 80 μs pulses **E**-**F** and longer pulses 160 μs **G-H**. Images were recorded with a high speed camera with an interframe time of 6 μs. Scale bars indicate 10 mm.

The pair of recorded images, I_0_ and I_1_, showed strong variations in brightness (Figure 2A+B). The second image of the image pair exhibited stronger luminescence increasing by up to a factor of 1.5–2.5 under anoxia, as compared to the fully aerated solution. The resulting integrated luminescence decay had brightness values ranging between 2.4% ± 0.076% and 8.0% ± 0.20%, which were substantially higher than the camera-specific noise level of 0.014%. In the anoxic cuvette, the absorption of photons by the sensor particle suspension significantly diminished the excitation light at the bottom of the cuvette, leading to a gradient in phosphorescence intensity (Figure 2C). To mitigate these variations that are unrelated to the O_2_ concentration, we applied a pixel-wise normalization of the emission intensity (eq 6). This postprocessing step compensated for a gradient of excitation light and resulted in integrated luminescence decay values that were uniformly distributed throughout the cuvettes (Figure 2D). The resulting normalized values ranged from 15 ± 1% to 44 ± 4%, i.e., a P_0_/P_100_ ratio of ∼3, which is in line with typical Stern–Volmer ratios of the O_2_ sensor material (e.g.,^18^, (29)) and values predicted by the model.

Subsequently, we explored the effects of varying pulse durations on the normalized, integrated luminescence decay values by increasing the laser pulse duration from 80 to 160 μs (Figure 2E-H). We observed that, although a longer pulse duration markedly increased brightness, the normalized, integrated luminescence yield value was reduced by approximately half. This suggests that the integrated luminescence decay remains constant and is unaffected by excitation time, as long as the excitation and emission of the luminophores is in equilibrium by the end of the laser pulse. Shorter pulses resulted in a more pronounced signal, as the integrated luminescence decay relative to the integrated excitation is stronger. However, reducing the pulse duration too much decreased the signal-to-noise ratio, as the number of photons reaching the camera chip decreased. Moreover, if the pulse duration is insufficient to reach the equilibrium of excitation and emission, the integrated luminescence decay will become a function of the pulse duration. Consequently, there is an optimal balance between pulse length and signal-to-noise ratio to maximize signal clarity and measurement accuracy for specific sensor materials.

**Figure 3:**
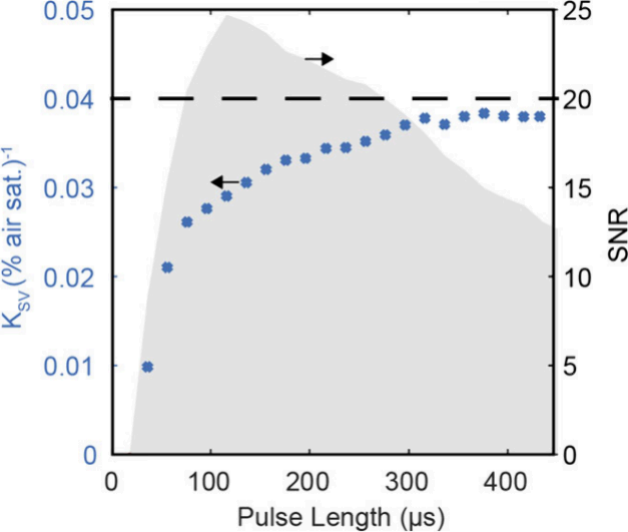
Response of the Stern–Volmer quenching constant (K_SV_, blue symbols) and the signal-to-noise ratio (SNR, gray area) for varying pulse lengths of the excitation source. The depiction indicates the optimal pulse duration of 76 μs - 256 μs, for which the signal-to-noise ratio is above 20. The cutoff of 20 was selected based on visual inspection of the raw images.

To identify the optimal pulse lengths, we conducted experiments using the oxic/anoxic cuvettes, while systematically varying the laser pulse durations from 61 to 500 μs in increments of 20 μs (Figure 3). We analyzed the resulting image pairs to calculate the Stern–Volmer constant (K_SV_) and to assess the signal-to-noise ratio (SNR) of the normalized integrated luminescence decay images. For the Stern–Volmer constant, we averaged values in the cuvette and applied eq 7 to estimate K_SV_ based on the two values for 100% air sat. and 0% air sat. Furthermore, the SNR was approximated by calculating the ratio of the average of the P_N_ over the standard deviation P_N_ within the cuvette. At shorter pulse lengths, the K_SV_ markedly decreased, likely due to the incomplete excitation of luminophores during short pulses. At a pulse length of 76 μs, K_SV_ stabilized with SNR peaking at 120 μs. However, K_SV_ continued to rise, attributed to the carry-over of phosphorescence from the first to the second frame (Figure S1A). Theoretically, adjusting the interframe time could mitigate this issue, but it would compromise the speed of the method. Therefore, we recommend setting the pulse length of the excitation light between 76 and 256 μs to achieve optimal SNR and image brightness, and camera inter frame time should be adjusted to at least 300 μs to obtain a stable K_SV_ with the used type of O_2_ indicator. Importantly, both calibrations and measurements should be performed with the chosen settings.

### Planar Optode Application

**Figure 4:**
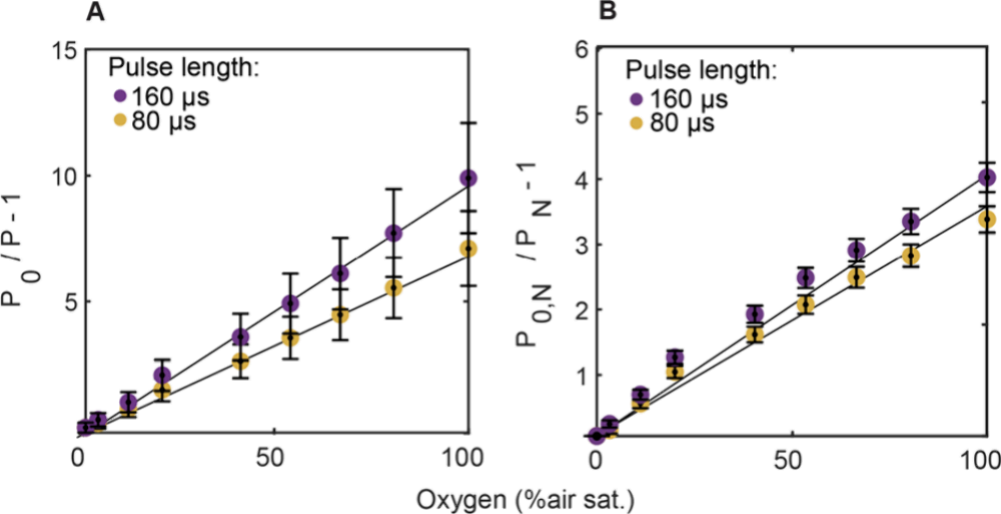
**A** Calibration of PtTFPP-based planar optodes using the frame-straddling method for imaging the integrated luminescence decay, P, as a function of O_2_ concentration. The Stern–Volmer constants are K_SV_ = 0.0710% air sat. ^–1^ (P_0_ = 0.22, R^2^ = 0.99) and K_SV_ = 0.105% air sat. ^–1^ (P_0_ = 0.23, R^2^ = 0.99) for 80 and 160 μs pulse length, respectively. **B** Calibration of the normalized integrated luminescence decay. The Stern–Volmer constants are K_SV_ = 0.0391% air sat. ^–1^ (P_0_ = 1.58, R^2^ = 0.99) and K_SV_ = 0.0458% air sat. ^–1^ (P_0_ = 0.63, R^2^ = 0.99) for 80 and 160 μs pulse length, respectively. Error bars in **A** and **B** denote standard deviation across the entire planar optode. Please note that the larger errors in **A** are related to inhomogeneous illumination (see Extended Discussion for further information).

We used the new imaging approach with PtTFPP-based planar O_2_ optodes^30^ to assess how the integrated luminescence decay changed as a function of O_2_ concentration. Experimentally this entailed adjusting the O_2_ levels within a cuvette equipped with a transparent planar optode on one side. Excitation of the planar optode was done with a high-power LED (LPS 3, ILA5150 GmbH) with a light-collimating lens. The resulting calibration curves showed linear Stern–Volmer plots (Figure 4), which indicates that despite high excitation light intensities no depopulation of the ground state of the luminophore occurred.^31^ However, it is important to note that the use of a high-speed camera, combined with the short interframe timing of approximately 7 μs, leads to a carry-over of phosphorescence from the first frame to the second frame (Figure S1C). We observed a 15% reduction in the K_SV_ value when using a 160 μs pulse duration, as compared to 80 μs. This is likely due to carry over in combination with an incomplete excitation of the sensor dye when using short pulses (Figure S1C+D). Still, the results for the planar optodes demonstrate a reliable calibration, despite the observed dependency on the pulse length. This was further confirmed through the calibration of other O_2_ indicator dyes, which vary in their sensitivity to O_2_ and have different emission spectra (see Figure S2 and Extended Discussion).

**Figure 5:**
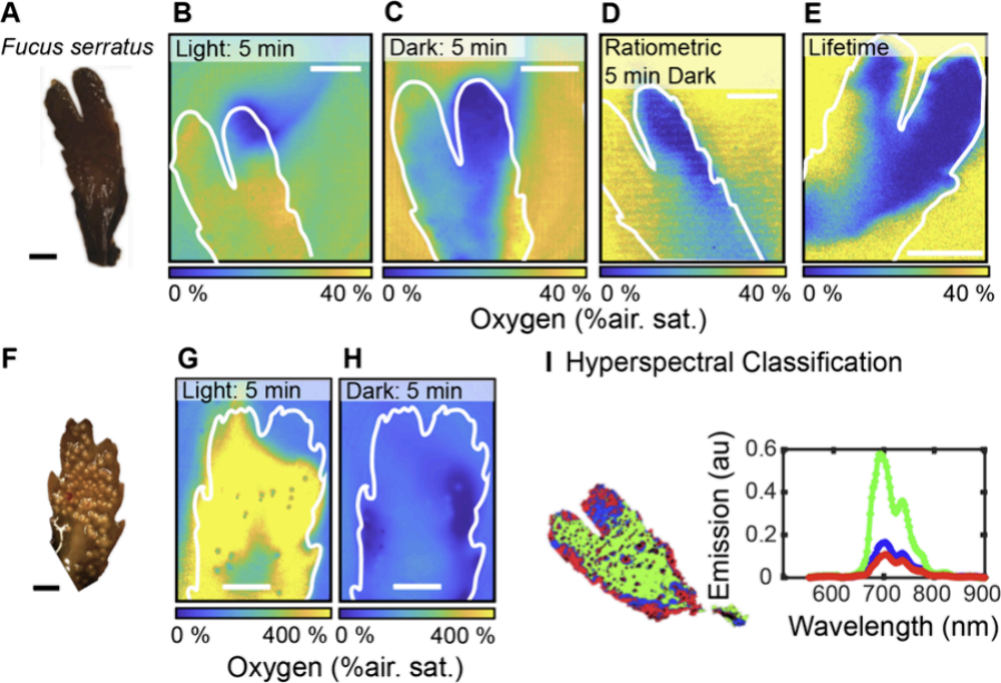
Variations in O_2_ saturation that result from O_2_ consumption and production of the brown alga *Fucus serratus*. Photo of a deteriorated fragment (**A**), and the corresponding O_2_ distribution under light (**B**) and dark (**C**) conditions, as quantified by the integrated luminescence decay method using frame straddling (see calibration in Figure 4). A laser pulse duration of 160 μs was applied and 50 images were recorded and averaged. Ratiometric luminescence intensity (**D**) and lifetime (**E**) imaging of the O_2_ distribution over the same sample under dark conditions. **F** Image of an intact fragment, and the corresponding O_2_ distribution of the intact fragment under light (**G**) and dark (**H**) conditions, as quantified by the integrated luminescence decay method. A pulse length of 194 μs was applied and 50 images were averaged. **I** Hyperspectral classification of the chlorophyll *a* distribution and the fluorescence emission in *Fucus serratus* after blue light excitation (fragment shown in **A-E**). Scale bar in all panels indicates 5 mm.

We subsequently employed the calibrated planar optode together with frame straddling-based imaging of the integrated luminescence decay to monitor the O_2_ dynamics in samples from the brown algae *Fucus serratus* (*Phaeophyceae*) under light dark conditions (Figure 5 and Figure S3 for experimental setup).^32^ We used two distinct fragments of this algae: one that appeared deteriorated with biofilm overgrowth in certain areas (Figure 5A-E), and another fragment that appeared intact (Figure 5F–H). First, the deteriorated algae sample was placed inside a custom-made chamber filled with seawater and with the calibrated planar optode on one side (see Methods). Initial O_2_ concentrations in the surrounding stagnant seawater were relatively low, i. e., ∼ 30% air sat. corresponding to 64.5 μmol O_2_ L^–1^ at experimental temperature and salinity. After 5 min light exposure, algal photosynthesis had increased the O_2_ levels to ∼40% air sat. over the surface of the algal thallus (Figure 5 B). The upper edge of the fragments showed substantially lower O_2_ levels, close to 0% air sat., indicative of intense O_2_ respiration. Chlorophyll (Chl) *a* in this area was either degraded by bacteria or absent due to the growth of the algae, which predominantly occurs at the algae tips.^33^ The O_2_ distribution on the surface was uneven, with small circular areas of reduced O_2_ concentration, most likely representing inert gas-filled vesicles in the algal tissue. In darkness, O_2_ levels over the algal thallus surface dropped to 0–5% air saturation; besides the lower right corner, where higher O_2_ levels were due to detachment of the algae from the planar optode (Figure 5B).

We then compared the frame straddling method with commonly used ratiometric and lifetime imaging techniques (see Methods) using the same algal sample, which revealed similar patterns of O_2_ consumption under dark conditions showing low O_2_ concentrations over the algal thallus surface and periphery reaching 0–50% air saturation (Figure 5D+E). Interestingly, we could resolve structures down to 400 μm in our O_2_ images based on quantification of the integrated luminescence yield with frame straddling, to ratiometric and lifetime imaging, where structures were in the millimeter range (see also Extended Discussion and Figure S4-Figure S5). However, it should be noted that this can also be related to a slight detachment of the *F. serratus* algae from the surface of the planar optode in the ratiometric and lifetime imaging setup.

Measurements on an intact fragment of *F. serratus* in light showed an immediate increase in O_2_ production, which reached levels of ∼400% air saturation within 5 min, corresponding to 860 μmol O_2_ L^–1^ (Figure 5F,G). This is in line with previous microsensor measurements that revealed O_2_ concentrations around *F. serratus* reaching up to 1,096 μmol O_2_ L^–1^.^34^ Additionally, small gas vesicles and/or crevices (cryptostomata) on the surface of *F. serratus* were observed as circular areas exhibiting reduced O_2_ concentrations, similar to the findings in the degraded fragment. Under dark conditions, the algae immediately started to respire O_2_ resulting in strong O_2_ depletion at the algae surface within a few minutes (Figure 5H).

Utilizing the quantification of the integrated luminescence decay with the frame straddling method in conjunction with planar optode-based imaging thus enables the monitoring of temporal variations of O_2_ concentration with high spatial resolution within submillimeter to centimeter applications (see Extended Discussion). The method is particularly strong at resolving high O_2_ concentrations, as the normalized luminescence yield exhibits a linear relationship in the Stern–Volmer plot (Figure 4), ensuring reliable measurements beyond air saturation. Although, *F. serratus* is strongly autofluorescent, as revealed by hyperspectral imaging (Figure 5I), no effects of autofluorescence were observed (see also Extended Discussion and Figure S6).

### Sensor Particle Application

We also tested the frame straddling method for imaging the integrated luminescence decay of dispersed O_2_ sensor nanoparticles for combined measurements of flow and O_2_ in a sensPIV setup.^35^ So far, sensPIV measurements have mostly been based on ratiometric imaging of sensor particles exhibiting an inert green reference fluorescence and an O_2_-dependent red luminescence matching the RGB-chip of a color camera.^36^ First, we evaluated the calibration of the sensor particles at dilutions of 1:1000 and 1:100 (based on a stock solution of 4 mg mL^–1^, see Methods). We further evaluated effects of varying magnifications (∼2 × 2 cm and ∼5 × 5 mm) in our frame straddling imaging setup. At low magnification, using laser pulse lengths of 56 and 156 μs, our observations revealed linear Stern–Volmer plots of the both the integrated luminescence decay and the normalized integrated luminescence decay as a function of O_2_ concentration. At higher magnifications, using a laser pulse length of 206 μs, the measurements were influenced by larger particle aggregates that skewed the calculation of the integrated luminescence decay (data not shown). Nonetheless, when calculating the normalized integrated luminescence decay, the signal stabilized and showed the expected linear response in the Stern–Volmer plot, where the quenching constant, K_SV_, only varied between 0.032 and 0.036 for laser pulse lengths of 56 μs, 156 and 206 μs, despite variations in magnification and sensor particle density (Figure S7).

**Figure 6:**
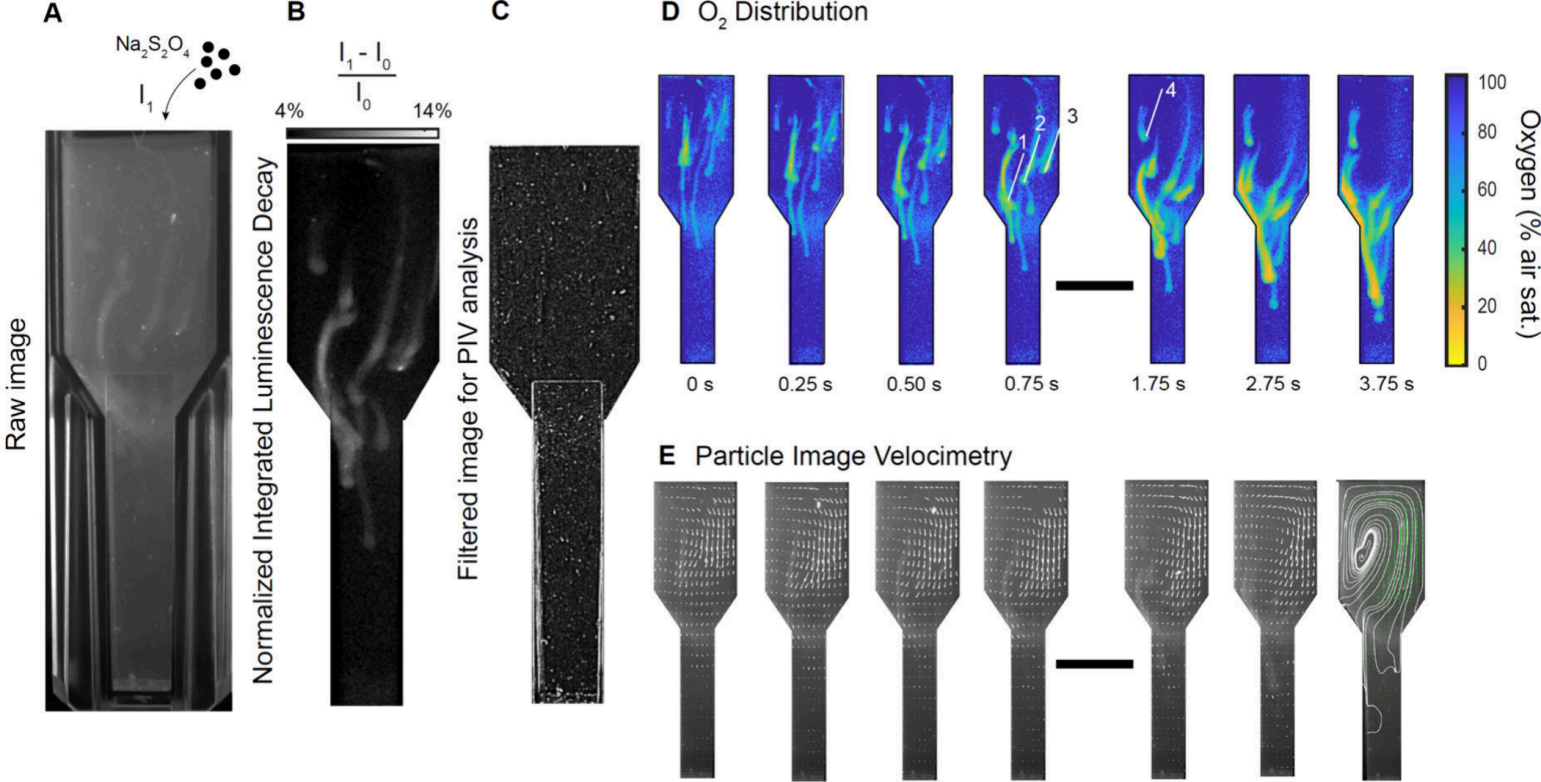
Combined O_2_ and PIV measurements (sensPIV) of the movement and reaction of sodium dithionite (Na_2_S_2_O_4_) granules with O_2_ in a water-filled cuvette, captured via frame straddling at 100 frames per second with a laser pulse length of 206 μs. **A** Raw image of the cuvette with slight changes in the brightness indicating variations in the O_2_ concentration upon addition of Na_2_S_2_O_4_. **B** Normalized integrated luminescence decay in a raw depiction, indicating strong O_2_ gradients within the cuvette. **C** Raw image after postprocessing and applying a suite of mathematical filters (see Methods). **D** The change of the O_2_ distribution in the cuvette with time for a total window of 3.75 s (see also Video S1). Numbers refer to specific granules selected for tracking and determination of O_2_ concentrations (see text). **E** Processed flow field in the cuvette, as indicated with white arrows. The last panel indicates the flow streamlines. In **A-C** the width of the cuvettes are 10 mm, in **D-E** the scale bar denotes 10 mm.

We then captured high-speed (100 fps) visualizations of the reaction of sodium dithionite (Na_2_S_2_O_4_), a chemical O_2_ scavenger often used for zero calibrations of O_2_ sensors, in air-saturated water (Figure 6 and Video S1). We added ∼10 Na_2_S_2_O_4_ granules to a cuvette with aerated water and imaged their movement and impact on the aqueous O_2_ content over time (Figure 6A). The recorded image pairs were processed to calculate the normalized integrated luminescence decay (Figure 6B). Additionally, the acquired images were processed to calculate the flow field through particle image velocimetry (Figure 6C; see Methods). Upon addition to the liquid, Na_2_S_2_O started to react with O_2_ instantaneous, leading to the formation of spheroids with reduced O_2_ concentrations (34–56% air sat.). The granules were sinking through the cuvette at velocities of 9–12.4 mm s^–1^ (Figure 6D; granule 1–3). As a result of the sinking, wakes with O_2_ concentrations of ∼65% air saturation were formed, which diminished progressively with distance from the spheroid through diffusive replacement of dissolved O_2_. As the sinking velocity gradually reduced from an average of 10.6 mm s^–1^ to 6.6 mm s^–1^, O_2_ started to deplete reaching values of 4.5% O_2_ air saturation.

Analysis of the fluid flow in the cuvette generated by the sinking Na_2_S_2_O_4_ granules yielded an instantaneous flow field of high quality, which required no further post processing besides an initial validation based on the range of velocities. The granules were primarily introduced on the right side of the cuvette, initiating a downward movement. The collective sinking of the granules and the associated entrainment effects resulted in a locally downward directed flow, through which the larger scale flow field within the cuvette was shaped. The flow on the right side reached values of around 5 mm s^–1^, which is in a similar range as the granule sinking velocity. The downward directed flow on the right side led to the creation of an upward directed flow on the left side. A vortex formed between upward and downward directed flows (Figure 6D+E), where a granule was caught in a suspension, likely due to counteracting flow forces balancing gravitational sinking (Figure 6D+E granule 4).

Our results demonstrate that utilizing the frame straddling methods for quantification of the integrated luminescence decay in sensPIV applications enables the monitoring of O_2_ concentration gradients with high temporal and spatial resolution (scales between 200 μm and several centimeter, see Extended Discussion). Our findings extend the applicability of sensPIV, presenting an alternative to ratiometric imaging that is less susceptible to background fluorescence and is adaptable to a wide range of optical sensing particles.

## Conclusions and Outlook

Using frame straddling for chemical imaging via a quantification of the integrated luminescence decay improves our ability to capture dynamically changing solute gradients with high temporal resolution over submillimeter to centimeter spatial scales. The method is compatible with a broad range of commercial scientific cameras and synchronizers and thus enables the advantages of lifetime imaging, while alleviating the requirement for using specialized (and expensive) time-domain or frequency-domain lifetime camera systems. We used the new method to visualize the development of chemical O_2_ gradients (both in planar optode and sensor nanoparticle applications) at recording frequencies of up to 100 Hz. In principle, with pulse durations of 250 μs, recording at frequencies as high as 2000 Hz is achievable provided the used camera has the required recording speed and the intrinsic limitation of the sensor-particles are considered (see Extended Discussion).

The calibration of the integral luminescence decay quantification via frame straddling as a function of O_2_ concentration is robust and yields linear Stern–Volmer relationship. While two-point calibrations at 100% and 0% air saturation are theoretically adequate, using a broader range of O_2_ concentrations significantly enhances analytical accuracy. Background fluorescence is effectively addressed by calculating the integrated luminescence decay, while the normalized integrated luminescence also compensates for inhomogeneous illumination. We introduced a model that predicts the integrated luminescence decay and calibration factors based on camera timings and luminophore lifetimes, which helps explain unexpected calibration responses due to potential carry-over effects from short interframe timings. Although these carry-over effects pose calibration challenges, they can also amplify the luminescence signal.

The new method advances flexible, chemical imaging, and can be further refined to improve resolution, accuracy, and the ability to capture microenvironmental chemical dynamics in various biotic and abiotic systems. While currently limited to lifetime proportional measurements, future enhancements, like using a second image pair with shifted timing would allow for direct lifetime estimates. The present study was based on using 1–5W laser diodes and LED’s limiting the application to sensor materials with luminescence lifetimes >5 μs. However, further optimization of sensor dyes in combination with fast cameras and stronger photodiodes would extend the applicability of the new method into ns-scale lifetime resolution and even better differentiation of background fluorescence from phosphorescent signals.

## Methods

### Sensor Particle Fabrication and Laser Light-Sheet Setup

#### PSMA Particles

Sensor preparation was done according to refs (37, 38). In short, we dissolved 100 mg of a styrene maleic anhydride copolymer (PSMA), 1.5 mg of Macrolex Fluorescence Yellow 10GN (MY, Macrolex Fluorescent Yellow 10GN, Lanxess, Köln, Germany), and 1.5 mg of the O_2_ indicator Platinum(II) meso-(2,3,4,5,6-pentafluoro)phenyl porphyrin (PtTFPP, Frontier Specialty Chemicals) in 10 g of tetrahydrofuran (THF). This solution was quickly introduced into 200 mL of vigorously stirred MQ water, followed by the evaporation of THF under an airstream. The suspension was concentrated at ∼60 °C until it reached a concentration of 4 mg mL^–1^. The sensor particles were then diluted in a ratio 1:100 and 1:1000 and transferred to the cuvettes.

#### PS–PVP Particles

Sensor preparation was done according to literature.^39,20^ In short, 526 mg poly(styrene-*block*-vinylpyrrolidone) polyvinylpyrrolidon (PS–PVP, Sigma-Aldrich) emulsion was mixed with a THF (30 mL) water (50 mL) mixture. Three mg PtTFPP and 3 mg MY were dissolved in 20 mL THF and added dropwise using a dripping funnel, into the rapidly stirred polymer emulsion. THF was removed via an N_2_ stream and the solution concentrated at 60 °C until it reached a concentration of approximately 4 mg mL^–1^.

Schematics of the used imaging setups are shown in Figure S3. To excite sensor particles, we used a 5W laser diode light source (450 nm) with a line optics emitting a ∼ 0.3 mm thick light sheet (Optolution GmbH). The laser was positioned ∼30 cm above the cuvettes for optimal excitation. Imaging within the light-sheet setup was performed with two monochromatic cameras: (i) A PIV camera (Optocam 2/80; Optolution GmbH) with a 6.6 mm 2.3 mega pixel sensor, capable of capturing double images at up to 160 Hz with an interframe time of τ_C_ = 62 μs (8-bit), (ii) a high-speed camera (Chronos 1.4; Kron Technology Inc.) with a 8 mm, 1.4 mega pixel sensor with a minimum interframe time of τ_C_ = 10 μs (12-bit) and capturing double images up to 400 Hz. The cameras were fitted with different lenses to adjust the magnification: A f/1.4, 17 mm lens (Xenoplan, Schneider-Kreuznach GmbH) was used for low magnification and a f/5.6, long distance microscope (k2 Distamax fitted with the CF1 objective, Infinity Inc.) for high magnification. To ensure only recording of the emission wavelengths, we mounted a 535 and 550 nm long-pass filter on the lenses. The laser and camera were triggered through a synchronizer (Optolution GmbH), which was controlled through the PIVlab toolbox^40^ installed in Matlab 2023b (Mathworks).

### Planar Optode Setup

Planar O_2_ optodes were fabricated as follows: 1 mg of platinum(II) meso-(2,3,4,5,6-pentafluoro)phenyl porphyrin (PtTFPP, Frontier Specialty Chemicals), 1 mg of MY (Macrolex Fluorescent Yellow 10GN, Lanxess, Köln, Germany) and 120 mg of polystyrene (PS, MW 192.000 g mol-1, Sigma-Aldrich) were dissolved in 1 g of toluene. Using a glass pipet, we applied the sensor mixture in front of a precleaned knife coating device set to 90 μm and evenly spread the mixture over a clean, dust-free polyethylene terephthalate (PET, Puetz Folien, Taunusstein, Germany) foil fixed onto a glass plate using a film of 70% ethanol. The coated foil was air-dried for 1 h and cured overnight in a heating cabinet at 50–60 °C. The transparent planar optode was mounted inside a 3D printed chamber with a microscope slide window on the front side. The planar optode was mounted on the glass slide, with the sensing dye-coated side oriented inward. *Fucus serratus* was collected from shallow waters in Øresund in Helsingør, Denmark (56.0308° N, 12.5921° E). The fragments of *F. serratus* were transferred into the chamber and positioned in direct proximity to the planar optode. Excitation of the planar optode was performed by using an ultra bright blue LED (LPS3, 450 nm; ILA5150 GmbH) with a light collimator installed in front of the LED to ensure homogeneous illumination. Imaging and synchronization was performed with the high-speed camera as described above.

The planar O_2_ optodes for testing different indicator dyes were fabricated by initially dissolving 5 mg of either PtTFPP (Frontier Specialty Chemicals), Pd(II) meso-(2,3,4,5,6-pentafluoro)phenyl porphyrin (PdTFPP, (Frontier Specialty Chemicals) or Pt(II) meso-tetra(4-fluorophenyl)tetrabenzo-porphyrin (PtTPTBPF, (Frontier Specialty Chemicals) in addition to 5 mg MY (Macrolex Fluorescent Yellow 10GN, Lanxess, Köln, Germany) and 175 mg of monocrystalline diamond powder (1–2 μm, Microdiamant AG) in 5 g of 10% (w/w) polystyrene (MW 192.000 g mol-1, Sigma-Aldrich) in chloroform (>99.5%, Alfa Aesar). These sensor mixtures were subsequently applied on PET foils (Puetz Folien, Taunusstein, Germany) using a precleaned 120 μm knife coater (Byk-Gardner GmbH) and allowed to air-dry overnight. Additionally, a planar optode to test for compensation of background fluorescence was prepared by creating a mixture of 5 mg MR (Macrolex Fluorescent Red G, Lanxess, Köln, Germany) and 175 mg diamond powder in 5 g of 10% (w/w) polystyrene in chloroform and applying this with a precleaned 120 μm knife coater to a PET foil, after which the coated foil was air-dried overnight.

Ratiometric imaging of luminescence intensity from the planar optode was performed with a color camera (Grasshopper 3, FLIR) according to (41). The RGB image was separated into the red and green channel, from which the R/G ratio was calculated (Matlab 2023b; Mathworks). Calibration was performed based on a two-point fitting of the Stern–Volmer equation. Luminescence lifetime imaging was performed using a CCD camera with a modulatable shutter (PCO.Sensicam/Sensimod; Excelitas AG) according to (21). Utilizing a custom-made trigger system, the planar optode was repeatedly excited for 40 μs. The shutter of the camera was first opened with delays of 41 μs and then with delays of 47 μs for 1 μs until a total integration time of 100 ms was reached. This procedure results in two images at different time points in the phosphorescence decay curve. The lifetime was then estimated by assuming a monoexponential decay of the phosphorescence and using the two recorded frames (F_1_ and F_2_, see (21) for details):
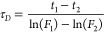
9where t_1_-t_2_ denotes the time between the two frames. Calibration was performed under varying O_2_ concentrations obtained by flushing solutions with N_2_ gas and addition of sodium sulfite (Na_2_SO_3_). The lifetimes of the planar optode were fitted by the equation τ_D_ = 1/(35.77·exp(-[O_2_]/56.3)+22.63) (R^2^ = 0.99), and this function was used in the model for the timing calculations.

### Hyperspectral Imaging

Hyperspectral imaging of algal thallus fragments was done using a SnapScan VNIR camera (imec-int.com) equipped with a color-corrected objective (Xenoplan XNP 1.4/23 CCTV-Lens, 400–1000 nm; schneiderkreuznach.com) and a 460 nm long-pass filter. For white referencing of the hyperspectral camera readout, 4 halogen lamps (OSRAM, DECOSTAR 51 ALU, 205 lm) at low intensity were used for even illumination of a 95% reflectance reference target (T95, Imec) to achieve comparable acquisition times as for luminescence imaging.^41,42^ Chlorophyll fluorescence was induced with a LED ring-light (8 × 405 nm LEDs; starlight-oe.com). Imaging was done using the system software (HSI Suite, IMEC) using the following settings; 64.15 ms integration time, 1 dB analog gain, 3 HDR frames, 3 HDR exposure ration, 2.5 maximum pixel blur. Corrected hyperspectral cubes were analyzed using the system software (HSI Studio, IMEC) for selecting regions with unique spectral properties and using the built-in software classifier tool to highlight areas with similar spectral patterns.

### Image Processing

In the planar optode experiments, the normalized phosphorescence yield was calculated for 50 images and subsequently averaged. We then applied the pixel-wise calibration to calculate the O_2_ in % air saturation. For the sensPIV measurements, two different processing steps were performed to calculate the O_2_ distribution and the velocity field from the raw images: (i) First, the normalized phosphorescence yield was calculated for each of a series of 500 image pairs. Subsequently, the calibration was applied for the specific laser pulse duration. To reduce the background noise of the recorded images, we calculated a pixel-wise 3-point moving average through the time series and applied a median filter with a 3 × 3-point kernel (see github repository: https://github.com/SoerenAhmerkamp/IntegratedLumiDecay). (ii) To determine the velocity field, we first reduced the brightness variation induced through the phosphorescence of the sensor particle by using a CLAHE filter with a 32 pixel window size to enhance local contrast. Subsequently, a high-pass filter with a 5 pixel kernel enhanced the appearance of particles, which improved the cross-correlation analysis. Finally, noise was removed from the image and low pass filtered by applying a Wiener2 filter with a 3 pixel window (the resulting image is shown in Figure 6C). After initial image processing, the displacement of particles was determined through a cross-correlation analysis using a Fast Fourier Transform deformation algorithm with an initial interrogation window size of 64 pixels with 50% overlap and a second pass with interrogation window size of 32 pixels. All image processing was performed in Matlab 2023b (Mathworks) in the PIVlab toolbox.^40^

## Data Availability

An example script to calculate the integrated luminescence decay is provided through github: https://github.com/SoerenAhmerkamp/IntegratedLumiDecay.

## Supporting Information Available

Figures showing phosphorescence response, calibration of planar optodes, schematics of the experimental setups, characterization of the planar optode applied for the algal experiments, further characterization of the planar optode, background fluorescence compensation, and calibration of O_2_ sensor nanoparticles for sensPIV. Extended Discussion section covering the precision, accuracy, and detection limit of the frame-straddling method, spatial and temporal resolution of the experimental setups, and hyperspectral scans of *Fucus serratus* fragments and background fluorescence compensation (PDF)
Reaction of sodium dithionate in saturated ambient water with the left panel showing the instantaneous O_2_ concentration and the right panel indicating the instantaneous flow field (AVI)

## Supplementary Material

se4c01828_si_001.pdf

se4c01828_si_002.avi
