# Supplementary material for: Novel Approach for Lifetime-Proportional Luminescence Imaging Using Frame Straddling
Source: ACS Sens. 2024 Oct 14;9(10):5531–40. doi: 10.1021/acssensors.4c01828 (PMC11519917; doi:10.1021/acssensors.4c01828)
Supplement: Supplementary file 1 — se4c01828_si_001.pdf [file se4c01828_si_001.pdf]

A novel approach for lifetime-proportional luminescence imaging using frame straddling

Soeren Ahmerkamp‡†* [1,2], Cesar O. Pacherres‡[3], Maria Mosshammer‡[3], Mathilde Godefroid[1], Michael Wind-Hansen[4], Marcel Kuypers[1], Lars Behrendt[5], Klaus Koren[4], Michael Kühl[3]

Correspondence: sahmerka@mpi-bremen.de

[1] Max Planck Institute for Marine Microbiology, 28359 Bremen, Germany, [2] Leibniz Institute for Baltic Sea Research, Rostock, Germany, [3] Marine Biological Section, Department of Biology, University of Copenhagen, Strandpromenaden 5, 3000 Helsingør, Denmark [4] Aarhus University Centre for Water Technology, Department of Biology, Aarhus University, 8000 Aarhus, Denmark [5] Science for Life Laboratory, Department of Organismal Biology, Program of Environmental Toxicology, Uppsala University, 75236 Uppsala, Sweden

Supplementary Figures

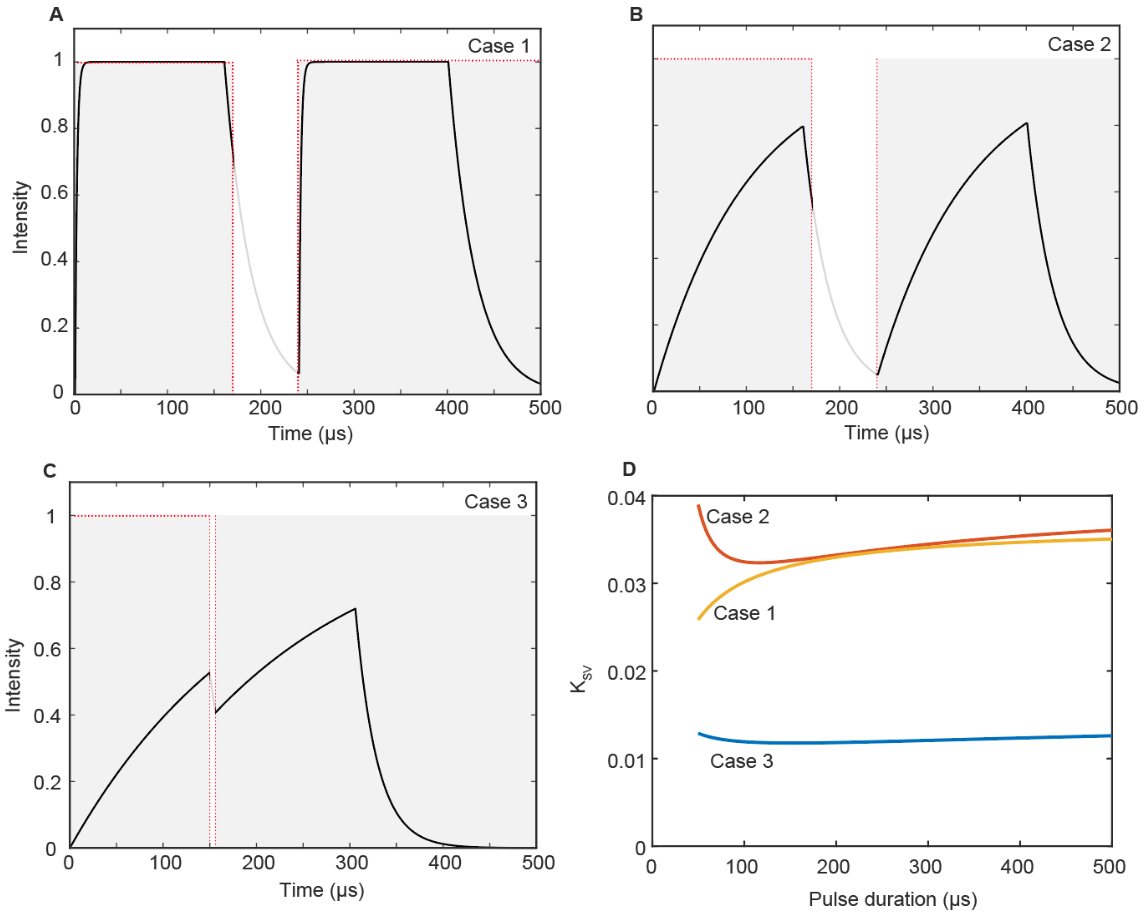

Figure S1. Phosphorescence response to frame straddling with varied timings. The timings are based on the cameras applied in this study and the relationship between O_2 concentrations and lifetime (see methods). **A** Ideally, luminophores are excited at the end of the first camera exposure, allowing for complete phosphorescence decay during the interframe period before being re-excited by the second laser pulse in the subsequent camera exposure. In the case depicted, there is a small carry-over effect. **B** When luminophore

excitation is slow, full excitation may not be reached. **A+B** depict timings used with the PIV camera (OptoCam, Optolution GmbH) and the sensor particles. **C** For slow excitation and brief interframe times (typical of high-speed cameras), phosphorescence carry-over results in emission pulses of different maximum intensities. **D** The response of the Stern-Volmer constant for the different cases calculated through model runs for 100% O_2 air saturation and 0% O_2 air saturation.

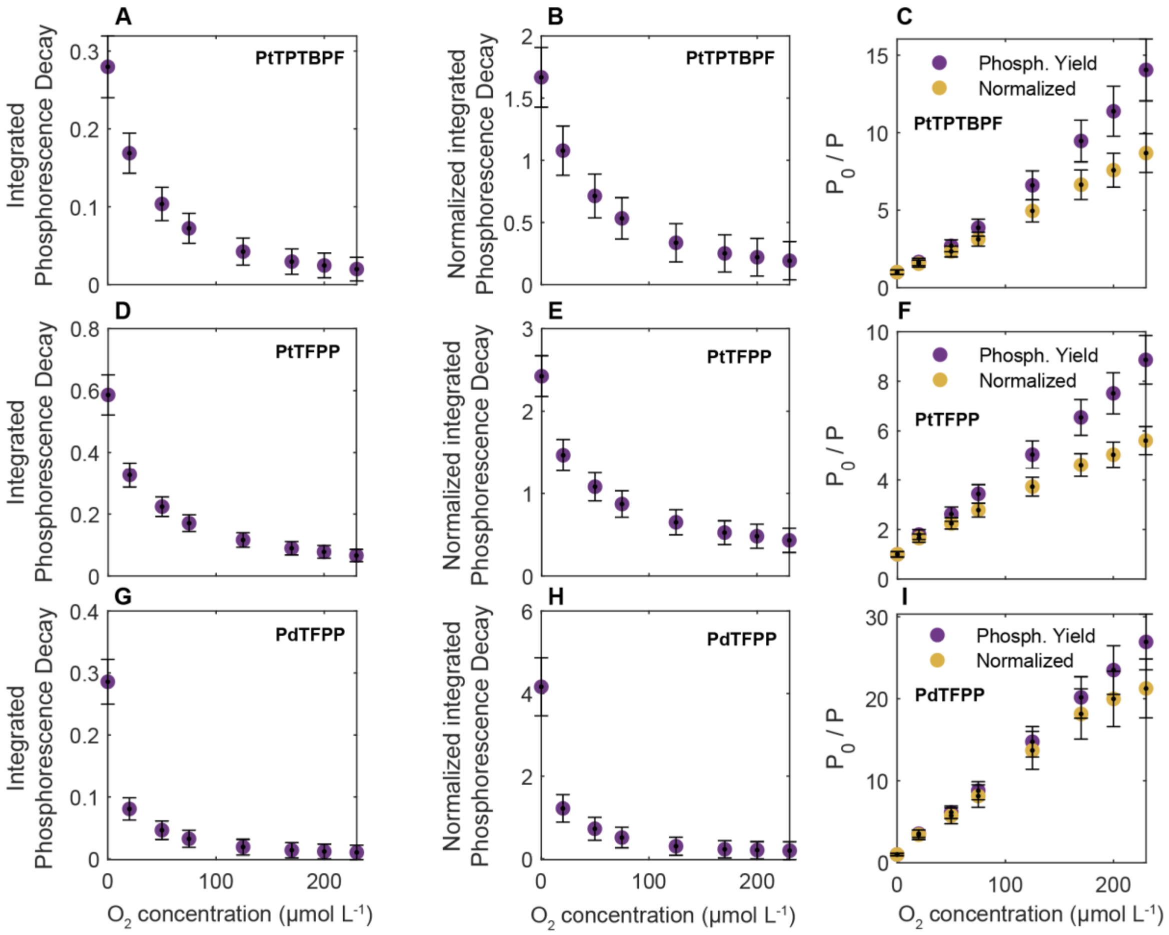

Figure S2. Calibration of planar optodes using the frame straddling method to quantify the integrated luminescence decay as a function of O_2 concentration for various O_2 indicator dyes (see also methods). Diamond granules were added to the planar sensor layer; this intensified brightness and LED pulses as short as 76 µs were sufficient for excitation. **A-C** Platinum complexes with benzoporphyrins exhibit good sensitivity to O_2 across the entire range, with notable differences between the dimmed and bright image pairs. However, the overall brightness was relatively low compared to other O_2 indicator dyes. Panels **D-F** reveal that palladium porphyrin complexes produce strong signals, especially at low O_2 concentrations, and their long lifetimes make them well-suited for use with the phosphorescence yield method. **G-I** Platinum porphyrin complexes maintain good sensitivity to O_2 throughout the range and display the strongest brightness. **A-I** Error bars represent the standard deviation, calculated from each pixel covering the respective planar optode.

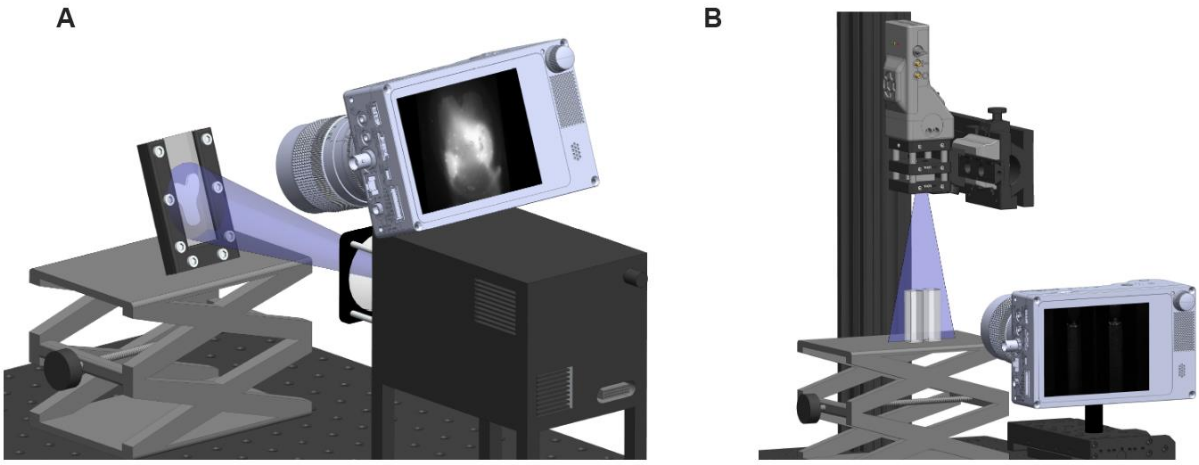

Figure S3. Schematics of the experimental setups. **A** The planar optode experiments as applied for data shown in Fig. 5 and **B** the light-sheet setup as applied for data shown in Fig. 2 and Fig. 5. Please notice that camera and light source were triggered via a synchronizer.

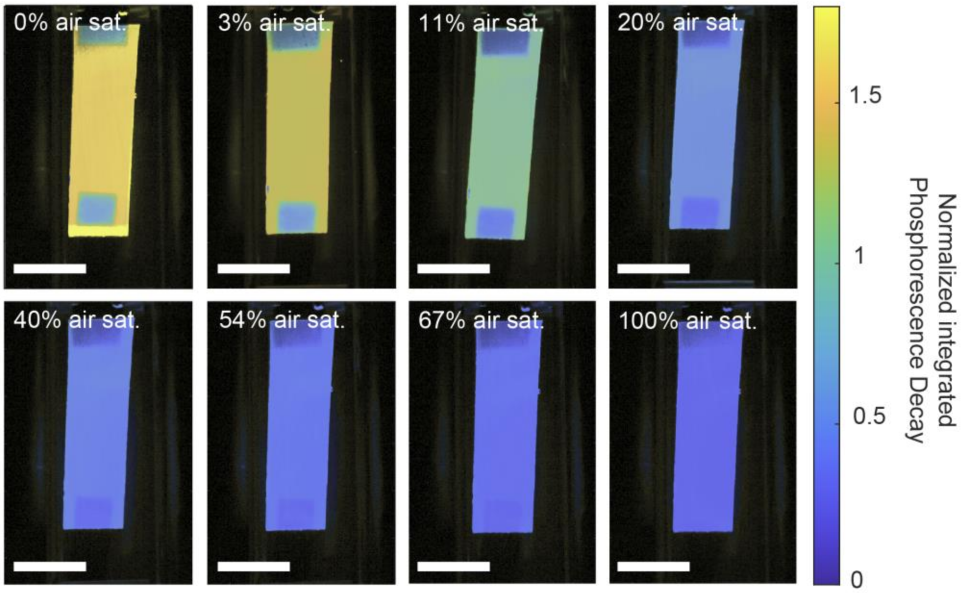

Figure S4. Characterization of the planar optode applied for the algae experiments. The planar optode demonstrates only little variability in the signal read-outs. Note that the sticky tape at the lower and upper and retains some O_2, making it visible at low O_2 concentrations. A scale bar of 10 mm is included for reference.

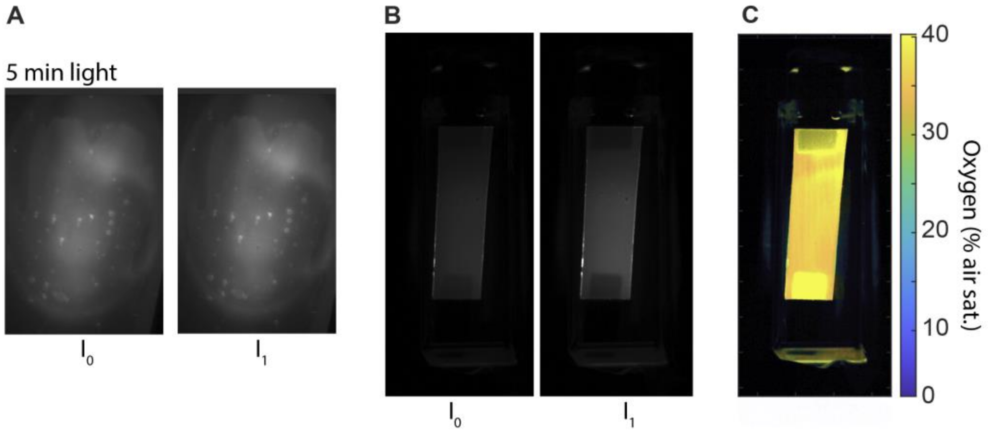

Figure S5. Further characterization of the planar optode used in the algae experiments. **A** brightness images of the results shown in Fig 5 B. **B** Brightness images of the calibration images depicted in Fig S4. **C** The planar optode was calibrated based on the data shown in Fig. S4. The axis scales are adjusted to range from 0% to 40% air saturation, i.e., similar to what is shown in Fig 5 B. While the average was 40% air saturation, we note that some gradients were observed as a result from the continuous degassing.

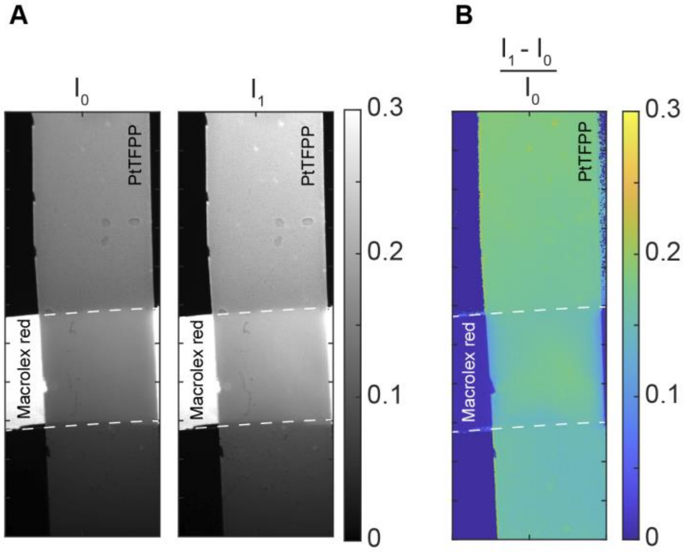

Figure S6. Background fluorescence compensation **A** Part of the oxygen-sensitive planar optode (PtTFPP) was covered with a foil containing Macrolex Red, which is a fluorescent dye that is excited in blue (max at ~500 nm) and emits in red (~600 nm) (see (39) for excitation spectra). The dimmed and bright image pair indicate variations of the oxygen-sensitive planar optode but little variations for Macrolex red. **B** The fluorescence induced through Macrolex Red is effectively compensated by calculating the normalized integrated luminescence decay from the image pair.

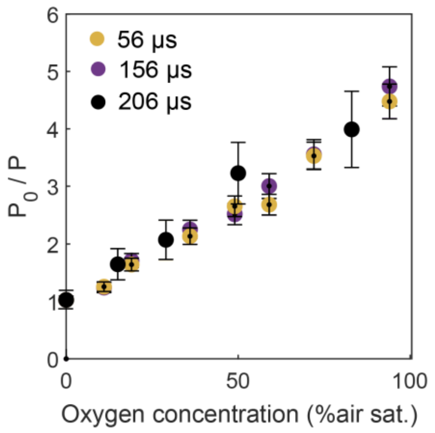

Figure S7. Calibration of O_2 sensor nanoparticles for sensPIV using the frame straddling method to quantify the O_2 dependent change of the integrated luminescence decay. The Stern-Volmer constants are K_{SV}=0.035 %air sat.$^{-1}$ (P_0=1.57, R^2=0.99), K_{SV}=0.034 %air sat.$^{-1}$ (P_0=0.51, R^2=0.99) and K_{SV}=0.032 %air sat.$^{-1}$ (P_0=0.55, R^2=0.99) for laser pulse lengths of 56 µs, 156 µs and 206 µs, respectively. We note that for the experiment with a laser pulse length of 206 µs, the magnification was increased and sensor particle density was reduced. Error bars denote the standard deviation within the selected region of interest.

Video S1. Reaction of sodium dithionate in O2-saturated ambient water. The left panel shows the instantaneous O2 concentration, while the right panel indicates the instantaneous flow field.

Extended Discussion

Precision, accuracy and detection limit of the frame-straddling method

We evaluated the applicability of the frame straddling method for quantification of the O_2 –dependent integrated luminescence decay with various O_2 indicator dyes in planar optodes. In this case the pulse-length was adjusted to 76 µs, as the addition of scattering diamond particles strongly enhanced the planar optodes brightness (*1*). Overall, we observed excellent performance across all tested O_2 indicators, although there was some variability in brightness and effectiveness at different O_2 concentrations (Fig. S2).

The precision was estimated by calculating the standard deviation of signal intensity across all pixels covering the planar optodes with varying indicator-dyes during calibration. The standard deviation derived from a single image pair is represented by the error bars in Figures 4 and Fig. S2. Overall, the precision is consistent for the normalized-integrated phosphorescent decay. For the integrated phosphorescent decay the signal intensities at low O_2 concentration exhibit stronger standard-deviations, which is likely due to inhomogeneous illumination that is not fully compensated.

Table S1: Normalized integrated phosphorescence decay values and corresponding standard deviations for various O_2 indicator dyes. The averaged values (averaged P_N) are derived from a sequence of 50 image pairs recorded within one second. SD refers to standard deviation.

Indicator		P_N	SD	Averaged P_N	SD
**PtTPTBPF**	100%	0.20	0.15	0.19	0.02
	0%	1.68	0.23	1.65	0.06
**PtTFPP**	100%	0.43	0.15	0.42	0.02
	0%	2.41	0.25	2.40	0.06
**PdTFPP**	100%	0.20	0.19	0.18	0.03
	0%	4.16	0.70	4.15	0.15

The precision of the frame-straddling method can be enhanced by averaging a sequence of images, which effectively reduces both the read noise and the shot noise associated with the camera chip. For example, when taking one of the image pairs for the calculation of PtTFPP the integrated phosphorescence decay value at 0% air saturation was 2.41 ± 0.25, whereas when taking the average of an image set of 50 images (recorded within one second), the value at 0% air saturation was 2.40 ± 0.06, implying that the precision increased by a factor of 4 (compare Table S1). Please notice, that the standard deviation is used as an indicator for the variability of signal read-outs, and the standard error of the mean is two orders of magnitude below the given values. Additionally, the image quality can be further improved by applying image filters, such as a median filter.

The accuracy of determining O_2 concentrations primarily depends on two factors: the concentration of O_2 and the properties of the applied O_2 indicator dye. At high O_2 concentrations, accuracy is lower compared to low O_2 concentrations. Additionally, the choice of the indicator dye plays a substantial role; for example, palladium-porphyrins provide much higher accuracy in predicting O_2 concentrations at lower levels compared to platinum-porphyrins, but it has significantly weaker accuracy at higher O_2 concentrations (see Fig S2). Based on the standard deviation of the integrated phosphorescence decay and an error propagation approach, we calculate the accuracy of the determined O_2 concentrations. For platinum-porphyrins the accuracy at 100% air saturation is approx. ± 5% air saturation corresponding to 12 µmol O_2 L^{-1} at experimental temperature and salinity, while at 0% air saturation the accuracy is approx.. ± 0.5 % air saturation corresponding to 1.2 µmol O_2 L^{-1} at experimental temperature and salinity. For palladium-porphyrins the accuracy at 100% air saturation is approx. ± 15% air saturation corresponding to 35 µmol O_2 L^{-1}, while at 0% air saturation the accuracy is approx. ± 0.2 % air saturation corresponding to 0.36 µmol O_2 L^{-1}.

The detection limit of the frame-straddling method is determined by the number of photons reaching the camera chip. In our setups, we adjust the laser pulses to a minimum duration between 76 µs and 256 µs, which we found optimal for the signal-to-noise ratio (refer to Fig. 3). It is important to note that increasing magnification will significantly reduce the photons captured by the camera, necessitating a stronger light source. We established a detection limit for phosphorescent decay at 5 µs in our setups. Although achieving

decay times below 1 μs is theoretically possible with stronger light sources and shorter laser pulses, this depends on high quantum yields from the indicator dyes and high quantum efficiencies of the camera chip.

The detection limit is also influenced by the specific O_2 indicator dyes used. Therefore, we have estimated intervals based on the precision of each sensor dye. We recommend using palladium-porphyrins within a range of 0% to 30% air saturation. Platinum porphyrins are effective across the entire range of O_2 concentrations and can also be utilized. However, it should be noted that at 100% air saturation, the measurement error is approximately ±5% air saturation. When extrapolating to 200% air saturation, this error increases to approximately ±15% air saturation. Platinum-benzoporphyrin planar optodes with an O_2-dependent emission in the NIR spectral range (780-800 nm) also worked well with the frame straddling method and exhibited strong differences between the recorded image pairs (Fig. S2 A-C), albeit their overall brightness was lower (and error margin higher), as compared to the palladium- and platinum-porphyrin based planar optodes. This is likely due to lower NIR sensitivity of the used camera system. The NIR emission property can however be advantageous, for example, when applied for subcutaneous O_2 monitoring of tissues (*2*,*3*).

Spatial and temporal resolution of the experimental setups.

The applied combination of camera, optics and light source, as well as the properties of the sensor-dye, determine the achieved spatial and temporal resolution. We succeeded in applying the method for field of views that were between 29 mm - 36 mm with a pixel resolution of 28 μm^{-1} per pix for the algae experiment and 49 mm – 79 mm with a pixel resolution of approximately 41 μm^{-1} per pix. The optics were similar in the ratiometric imaging and lifetime imaging setups. The smallest resolvable structures were of about 400 μm in the algae experiments (the vesicles) and 200 μm in the sodium dithionite experiment (the wake of reduced O_2 concentrations). For the algae, the ratiometric and lifetime imaging approaches only resolved structures in the milimeter range. It is important to note that ratiometric imaging necessitates the use of color cameras equipped with a Bayer filter (RGGB), which reduces the resolution by a factor of two. While larger magnifications are in principle possible, it would require stronger light sources and then other limitations, such as the depopulation of the ground state associated to increasing excitation light intensities, must be considered. We note that the high-speed recording of the O_2 dynamics in solution upon addition of O_2-scavenging sodium-dithionite crystals is not possible with conventional lifetime imaging and ratiometric imaging approaches due to their lower image acquisition times.

In high-speed imaging, the delayed diffusion of O_2 into the polymer of the sensor particles introduces inherent limitations in both spatial and temporal resolution. For the sensor particles used in the sodium-dithionite reaction experiments, the median diameter was d=~300 nm resulting in the diffusion timescale of [illegible]$=(0.5d)^2$ / (2D) ~ 10 ms, when considering a diffusion coefficient of ~$1 \cdot 10^{-12}$ m^2 s^{-1} for PSMA (4). This timescale is comparable to the frame rate of the camera (100Hz = 10 ms), indicating that faster recordings would not yield

improved temporal resolution of the reaction. Further, the spatial resolution is influenced by the movement of sodium-dithionite granules within the 10 ms time window. The granules were observed to sink at a rate of 12.4 mm s^{-1}, leading to a displacement of approximately 120 µm between frames (10 ms intervals). This displacement sets the upper limit for spatial resolution in these experiments. It is important to note that these calculations are conservative, as they assume complete diffusive quenching of the sensor particles. The temporal resolution of 10 ms surpasses that of conventional lifetime and ratiometric imaging, which typically is in the range of ~50 ms to several seconds.

Hyperspectral scans of *Fucus serratus* fragments and background fluorescence compensation

The O_2 fluxes of the intact *Fucus serratus* fragments were -2.6 mmol O_2 m^{-2} h^{-1} under dark conditions and 10.3 mmol O_2 m^{-2} h^{-1} under light conditions. These fluxes are substantially higher compared to those of the degraded fragment and slightly exceed previous measurements that were performed within the diffusive boundary layer (5). To confirm the degradation status of the fragments, we performed hyperspectral scanning of the degraded and intact fragment under blue light excitation (Fig. 5I). The degraded fragment contained Chl *a*, but closer inspection revealed that the Chl *a* fluorescence at the margins of the algae was substantially reduced and the peaks were shifted from 690 nm towards higher wavelengths indicating degradation. When classifying the Chl *a* fluorescence into different classes representative of the Chl *a* quality and quantity, we observed a good match between regions of degraded chlorophyll *a* and low O_2 levels at light conditions (compare Fig. 5 B+I). For the intact fragments, we observed substantially more pronounced Chl *a* bands. It is important to mention, that the fluorescence from Chl *a* would result in an underestimation of the O_2 levels in ratiometric imaging approaches, which we did not observe with the frame straddling method indicating an effective background fluorescence compensation. To further a good background fluorescence compensation, we performed test experiments with the fluorescent dye Macrolex Red which we coated onto a foil and placed behind a PtTFPP planar optode (Fig S4 **a-c**). The strong emission of Macrolex Red was visible in the raw luminescence images, but was compensated for when calculating the integrated luminescence decay.

Bibliography

1. M. Mosshammer, M. Strobl, M. Kühl, I. Klimant, S. Borisov, and K. Koren: Design and application of an optical sensor for simultaneous imaging of pH and O2 with low cross-talk. *ACS Sensors* 1: 681-687 (2016). doi: 10.1021/acssensors.6b00071

2. S. M. Borisov, G. Nuss, I. Klimant, Red light-excitable oxygen sensing materials based on platinum(II)

and palladium(II) benzoporphyrins. *Anal. Chem.* **80**, 9435–9442 (2008). doi: 10.1021/ac801521v

3. M. Kühl, D. A. Nielsen, S. M. Borisov, *In-vivo* Lifetime Imaging of the Internal O_2 Dynamics in Corals with NIR-emitting Sensor Nanoparticles *ACS Sensors* doi: 10.1021/acssensors.4c01029 (2024),.

4. Kucukpinar, E., & Doruker, P. (2003). Molecular simulations of small gas diffusion and solubility in copolymers of styrene. Polymer, 44(12), 3607-3620. doi: 10.1016/S0032-3861(03)00166-6.

5. K. Spilling, J. Titelman, T. M. Greve, M. Kühl, Microsensor Measurements of the External and Internal Microenvironment of *Fucus vesiculosus* (phaeophyceae)[1]. *J. Phycol.* **46**, 1350–1355 (2010). doi: 10.1111/j.1529-8817.2010.00894.x
